# Advances and future perspectives of kidney organoid technology in renal disease research and clinical translation

**DOI:** 10.3389/fmed.2026.1815902

**Published:** 2026-04-29

**Authors:** Zichun Zhao, Kuan Tong, Kunfei Deng, Hongjie Liu, Yu Li, Danni Wang

**Affiliations:** 1 Department of Critical Care Medicine, the First Hospital of China Medical University, Shenyang, China; 2 Department of Nephrology, the First Hospital of China Medical University, Shenyang, China; 3 Department of Pancreatic-Biliary Surgery, the First Hospital of China Medical University, Shenyang, China; 4 Department of Cardiac Surgery, the First Hospital of China Medical University, Shenyang, China

**Keywords:** clinical translation, dialysis therapy, kidney organoid, organ-on-chip, precision medicine, renal disease

## Abstract

Kidney organoids, as three-dimensional *in vitro* models that recapitulate the structural and functional features of the human kidney, have made significant strides in recent years in elucidating the mechanisms of renal diseases, drug screening, and personalized therapeutic evaluation. This review provides a comprehensive overview of the current technologies for kidney organoid generation, their applications in renal disease research and treatment, and the engineering and translational challenges that remain. Particular focus is placed on recent advances in vascularization strategies, integration with bioprinting and organ-on-chip platforms, simulation of the immune microenvironment, and long-term culture techniques. We further discuss key bottlenecks in clinical translation, such as large-scale production, data reproducibility, and ethical and regulatory considerations. Finally, we highlight emerging trends in the convergence of kidney organoids with precision medicine, microfluidic systems, and biosensing technologies. Kidney organoids are poised to drive innovation in biotechnology, regenerative medicine, and pharmaceutical development, providing a powerful platform for the precise diagnosis and treatment of renal diseases.

## 1 Introduction

Renal diseases, particularly chronic kidney disease (CKD) and end-stage renal disease (ESRD), have emerged as major global public health challenges (1). According to statistics from the World Health Organization, approximately 8–10% of the global population suffers from varying degrees of kidney dysfunction. With the ongoing trends of population aging, and the rising prevalence of diabetes and hypertension, the incidence and burden of renal diseases are steadily increasing, severely impacting patient quality of life and the sustainability of healthcare systems worldwide (2, 3). Currently, the primary therapeutic strategies for kidney diseases include pharmacological treatment, dialysis, and kidney transplantation. However, these conventional approaches face significant limitations, including a lack of therapeutic targets due to poorly understood CKD pathogenesis, the incomplete replacement of renal function by dialysis, severe shortages of donor kidneys, and complications related to immune rejection (4). Therefore, there is an urgent need to develop *in vitro* models that more accurately recapitulate the physiological and pathological processes of the human kidney, to facilitate mechanistic research, drug screening, and the exploration of alternative therapies.

Organoids are three-dimensional tissue-like structures derived from stem cells that can self-organize into miniature models recapitulating key architectural and functional features of native organs under defined microenvironmental conditions. Compared with traditional two-dimensional cell culture systems, organoids provide greater structural complexity, multicellular interactions, and physiological relevance, enabling a more faithful reproduction of *in vivo* tissue microenvironments and disease processes (5). In recent years, kidney organoid research has advanced rapidly through the convergence of stem cell biology, developmental nephrology, three-dimensional culture systems, and tissue engineering (6). Importantly, this progress was established by several landmark primary studies. Early experimental work demonstrated that human pluripotent stem cells could be directed toward nephron progenitor fates and assembled into nephron-like organoids that model aspects of kidney development and injury (7). Soon thereafter, human induced pluripotent stem cell-derived kidney organoids containing multiple renal lineages were generated, providing a platform that more broadly recapitulated human nephrogenesis (8). Further advances extended these systems toward higher-order kidney organogenesis by incorporating developmental patterning processes related to ureteric bud formation and branching morphogenesis (9). In parallel, CRISPR-based kidney organoid models further showed that these systems could be applied to study human kidney disease mechanisms in a genetically tractable manner (10). This review systematically summarizes current strategies for kidney organoid generation, their applications in renal disease modeling, explorations of their integration into renal failure treatment, and potential directions for clinical translation. The goal is to provide a theoretical and technological foundation for kidney disease research, drug development, and personalized medicine.

## 2 Fundamental theories of organoids in renal disease research

Research and application of kidney organoids are grounded in a deep understanding of renal developmental biology. The advancement of organoid technology stems not only from mimicking kidney embryogenesis but also from integrating diverse engineering strategies and differentiation protocols to reconstruct miniature kidney models with structural and functional features in a three-dimensional environment. Different construction approaches—such as directed differentiation of pluripotent stem cells, self-organizing cultures, and chip- or scaffold-assisted systems—offer distinct advantages in structural control, cellular diversity, and functional maturation.

**Figure 1:**
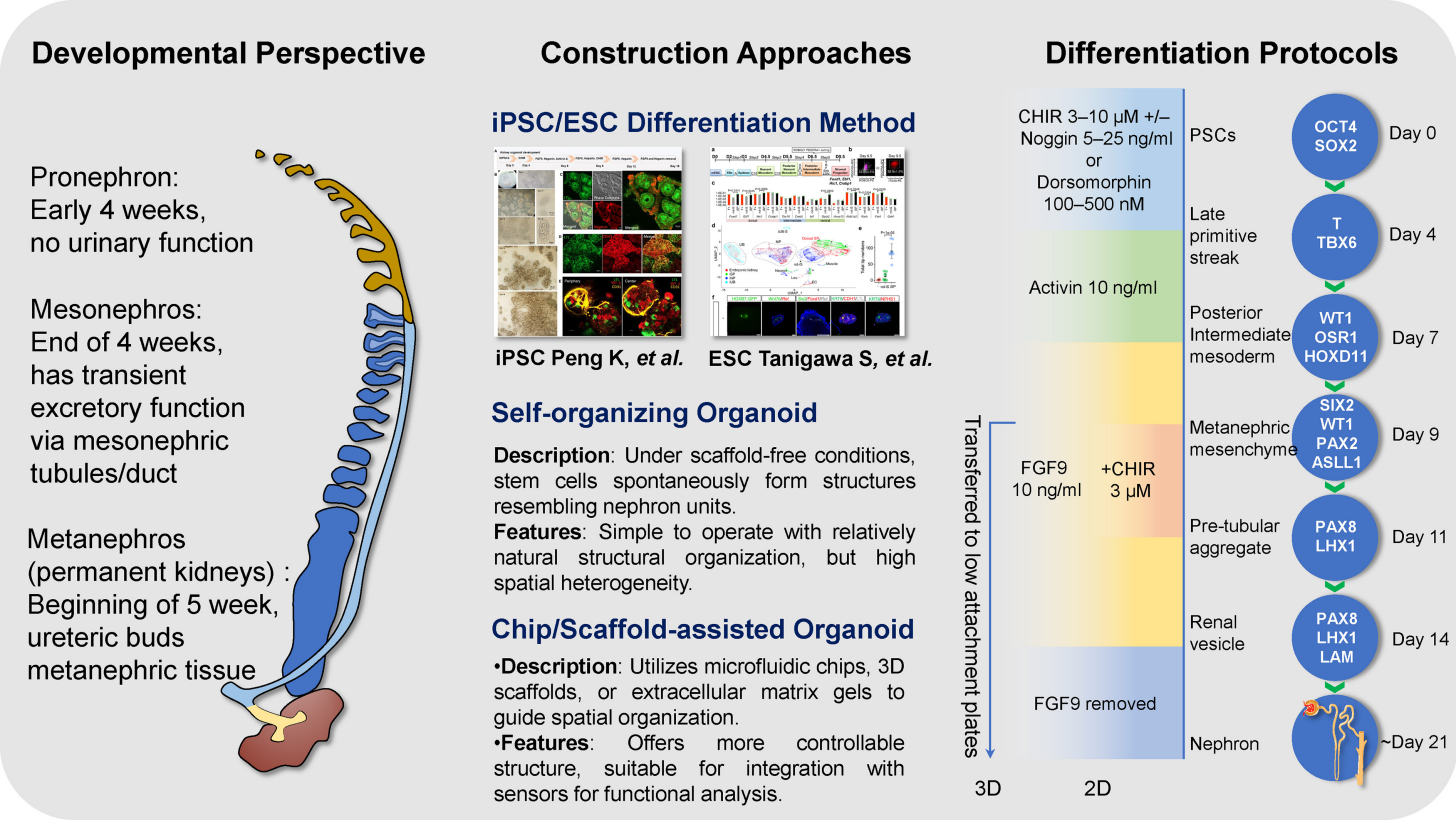
iPSC differentiation method reproduced from “Generation of kidney organoid from human induced pluripotent stem cells” by Kexin Peng, Wanqin Xie, Tingting Wang, Yamei Li, Jean de Dieu Habimana, Obed Boadi Amissah, Jufang Huang, Yong Chen, Bin Ni and Zhiyuan Li, licensed under CC BY 4.0. ESC differentiation method reproduced from “Mouse ESC-derived SPs support the generation of the complex kidney structure” by Shunsuke Tanigawa, Etsuko Tanaka, Koichiro Miike, Tomoko Ohmori, Daisuke Inoue, Chen-Leng Cai, Atsuhiro Taguchi, Akio Kobayashi and Ryuichi Nishinakamura, licensed under CC BY 4.0.

Embryonic origin, construction strategies, and differentiation workflow of kidney organoids. The figure summarizes the developmental and methodological basis of kidney organoid generation (Figure 1). Kidney development progresses from the pronephros (non-functional, ~week 4), to the mesonephros, which provides a transient excretory function through mesonephric tubules connected to the mesonephric duct, and ultimately to the metanephros (permanent kidney, ~week 5), in which the ureteric bud arises from the mesonephric duct and induces formation of the definitive collecting system. Organoid construction methods include directed differentiation of human pluripotent stem cells (hPSCs, including iPSCs and ESCs), self-organizing organoids under scaffold-free conditions, and chip/scaffold-assisted systems that improve structural control. The representative differentiation workflow is based on the original hPSC-derived kidney organoid study by Morizane et al. (7) and the subsequent detailed protocol reported by Morizane and Bonventre (11), showing stepwise transitions from PSCs to nephron-like structures over ~21 days guided by key signaling molecules (e.g., CHIR, Activin A, FGF9) and lineage markers (e.g., OCT4, T, WT1, PAX8).

This figure provides the developmental and methodological context for pluripotent stem cell-derived kidney organoids discussed in Section 2; tissue-derived renal tumor organoids represent a distinct platform and are addressed separately in later sections.

### 2.1 Development of organoid technology and its application in kidney disease research

Organoid technology represents a major breakthrough in the field of regenerative medicine, offering new opportunities for the study of kidney diseases (12). Traditional approaches such as animal models and two-dimensional (2D) cell culture systems have made important contributions to nephrology research but are limited by species-specific differences and the inability to recapitulate the complex *in vivo* microenvironment (13). Organoids, as three-dimensional (3D) multicellular clusters, can mimic tissue and organ architecture and function through the controlled application of growth factors *in vitro*, thereby overcoming many of the limitations associated with conventional methods.

Over the past decade, multiple renal organoid-related platforms have been established from distinct biological sources. Among them, human pluripotent stem cell (hPSC)-derived kidney organoids constitute the principal developmental platform for modeling nephrogenesis and non-neoplastic kidney disease. When combined with genome editing technologies, these hPSC-derived systems have become a powerful tool for investigating genetic kidney disorders and disease mechanisms. In contrast, tissue-derived renal epithelial organoids and renal tumor organoids represent separate systems with different biological purposes and translational applications. As previously noted, the incidence of CKD is rising rapidly, driven by increasing rates of diabetes and cardiovascular diseases. Dialysis significantly impacts patients’ quality of life, and the shortage of donor kidneys continues to hinder transplantation. The advent of kidney organoid technology brings renewed hope for addressing these challenges. By recapitulating renal development and disease processes, organoids provide valuable insights into the pathophysiological mechanisms of kidney disorders and a theoretical foundation for the development of novel therapies. Moreover, studying the signaling pathways involved in kidney organogenesis using organoid models may reveal potential therapeutic targets, paving new paths for the treatment of renal diseases (14).

### 2.2 Construction and optimization of kidney organoids

The construction of kidney organoids is grounded in the principles of developmental nephrology. Over the past decade, protocols have been established to derive two primary types of kidney organoids from pluripotent stem cells: nephron organoids and ureteric bud organoids (15, 16). The induction of kidney organoids from human pluripotent stem cells (hPSCs) typically involves multiple stages, beginning with the directed differentiation into intermediate mesoderm, followed by three-dimensional culture to promote self-organizing nephrogenesis, ultimately leading to the formation of structured organoids (17).

Multiple factors influence the quality and functionality of kidney organoids during their generation. Cell density is a critical determinant of organoid formation. Studies have shown that when using urine-derived stem cells, an optimal seeding density of 5,000 cells per well yields well-organized organoids with minimal cell death (18). The extracellular matrix (ECM) also plays a vital role by modulating cell signaling pathways and fate decisions. Specific ECM components can enhance the organoid microenvironment, thereby improving differentiation efficiency and functional maturation (19). Moreover, fine-tuning the culture conditions—such as extending the exposure time of hPSCs to a three-dimensional microenvironment and incorporating specific renal inductive signals—can render the transcriptomic profile of generated organoids more similar to that of fetal human kidneys (20).

The successful generation of kidney organoids relies on recapitulating human renal development through directed differentiation and spatial organization in 3D culture systems. Core components include the selection of appropriate stem cell sources, optimization of differentiation protocols, establishment of supportive structural systems, and evaluation of functional maturity. The following sections systematically elaborate on current methodologies and emerging directions in the construction of kidney organoids from three critical perspectives.

Challenges such as heterogeneity, immaturity, lack of vasculature, batch-to-batch variability, and scalability issues continue to limit reproducibility and functional relevance. Addressing these obstacles demands both biological and engineering solutions, from refining differentiation protocols and co-culture strategies to integrating automation and biosensing technologies. Table 1 summarizes the key technical bottlenecks in kidney organoid research and outlines representative strategies aimed at enhancing consistency, maturity, and functionality across experimental settings.

**Table 1:** Technical challenges and strategies for quality control in kidney organoids.

Challenge	Description	Strategies
Heterogeneity (68, 77)	Organoids vary in size, structure, and cell composition across batches.	Use of single-cell RNA-seq and spatial transcriptomics for profiling; standardization of cell input number and differentiation timing.
Immaturity (41, 64)	Organoids often resemble fetal kidney tissue rather than adult functional nephrons.	Prolonged culture, addition of maturation signals (e.g., retinoic acid, cAMP), co-culture with vascular/endothelial cells.
Lack of vasculature (67, 69)	Poor oxygen/nutrient diffusion leads to necrosis and limits size/function.	Vascular co-culture, transplantation into animal models, perfusion bioreactors, and stirred-bioreactor culture systems that enhance glomerular vascularization (71).
Batch-to-batch variability (78, 79)	Variations in reprogramming efficiency, medium components, or manual handling.	Automation of organoid generation; use of chemically defined media.
Functional assessment (80, 81)	Difficult to evaluate functional maturity (e.g., filtration, reabsorption).	Incorporation of real-time biosensors, microfluidic urine collection, electrophysiology assays.
Disease progression modeling	Difficult to reproduce the transition from acute kidney injury to maladaptive repair and chronic kidney disease progression in conventional short-term organoid systems.	Repeated injury paradigms, longitudinal follow-up of repair responses, and integration of fibrosis-associated molecular readouts to capture AKI-to-CKD transition features (82).
Scalability (41, 83)	Difficult to maintain quality during scale-up for high-throughput use.	Use of bioreactors, standardized microfabricated chips, parallelized differentiation platforms and delta-wing stirred bioreactor-based scalable production systems (71).

#### 2.2.1 Stem cell sources and differentiation induction strategies

Currently, the primary stem cell sources used for kidney organoid construction are iPSCs and ESCs, with iPSCs being widely favored due to their flexible origin and potential for personalized disease modeling. The differentiation process aims to recapitulate the natural developmental trajectory from mesoderm to renal progenitor cells, which relies on the temporal activation of multiple signaling pathways.

The representative differentiation timeline is based on the original hPSC-derived kidney organoid study reported by Morizane et al. (7) and the subsequent detailed protocol published by Morizane and Bonventre (11), showing stepwise transitions from PSCs to nephron-like structures over ~21 days guided by key signaling molecules and lineage markers. Kidney organoid generation begins with culturing human pluripotent stem cells (hPSCs) under feeder-free conditions (e.g., using ReproFF2 or StemFit Basic media) to maintain their undifferentiated state. Cells are then seeded at approximately 50% confluency in 24-well plates and induced towards posterior intermediate mesoderm using a basal differentiation medium supplemented with the GSK-3β inhibitor CHIR99021 (3–10 μM, adjusted according to cell line). BMP4 pathway inhibitors such as Noggin or Dorsomorphin can be added to enhance induction efficiency if necessary. After approximately 4 days, cells form loosely dense clusters indicative of successful posterior intermediate mesoderm induction, confirmed by the expression of markers such as WT1 and HOXD11.

Next, cells are treated with Activin A (10 ng/mL) for 2–3 days, followed by fibroblast growth factor 9 (FGF9, 10 ng/mL) for an additional 2 days, yielding a high-purity population (80–90%) of nephron progenitor cells (NPCs) characterized by expression of SIX2, PAX2, and WT1. NPCs can then be further differentiated into kidney organoids through either 2D or 3D culture approaches:

In 2D culture, NPCs are exposed to CHIR99021 (3 μM) and FGF9 (10 ng/mL) for 2 days to form pretubular aggregates expressing PAX8 and LHX1, followed by 2–3 days of FGF9 alone to develop renal vesicle-like structures. Growth factors are then withdrawn for 1 week, leading to segmented nephron structures containing podocytes, proximal tubules, loops of Henle, and distal tubules.

In 3D culture, NPCs are transferred to ultra-low attachment 96-well round-bottom plates at approximately 100,000 cells per well and subjected to sequential treatments with CHIR99021 plus FGF9, FGF9 alone, and finally growth factor withdrawal, resulting in spherical kidney organoids approximately 0.5 mm in diameter comprising multiple nephron-like structures.

These organoids can be used for immunostaining of markers such as SIX2, PODXL, and LTL, drug toxicity assays (e.g., cisplatin-induced injury monitored by KIM-1 expression), or disease modeling using patient-derived iPSCs or CRISPR-mediated gene editing to study inherited renal disorders. Throughout the protocol, close monitoring of cellular morphology is essential, and CHIR99021 concentration and seeding density must be optimized according to different hPSC lines. Maintaining the freshness of culture media and growth factors is also critical to enhance experimental reproducibility.

#### 2.2.2 Three-dimensional culture systems and structural support strategies

To enable differentiated cells to form three-dimensional tissue structures characteristic of organoids, appropriate 3D culture systems that provide physiological microenvironmental support are essential. Commonly used scaffolding systems include ECM embedding with hydrogels such as Matrigel (21), air–liquid interface (ALI) culture (22), as well as assistance from bioprinting (23) and microfluidic platforms (24). Matrigel offers a basement membrane-like ECM architecture that helps maintain cell polarity and spatial organization; the ALI system enhances oxygen exchangeand is beneficial for promoting tissue maturation. More recently, emerging technologies such as microfluidic chips and 3D bioprinting provide precise control over organoid morphology, spatial arrangement, and nutrient supply, thereby improving biomimicry and reproducibility of the models.

Moreover, advances in biomaterials have greatly expanded the possibilities for organoid scaffold construction. Natural polymers such as gelatin (25), silk fibroin (26), and alginate (27) have been employed in renal tissue engineering to fabricate biodegradable 3D scaffolds with tunable mechanical properties, offering both mechanical support and spatial cues conducive to organoid formation.

#### 2.2.3 Functional and maturation assessment of organoids

Several studies have successfully generated kidney organoids that exhibit fundamental functions, with their internal structures revealing glomerulus-like structures, proximal tubules, and collecting duct regions. These organoids express a variety of functional markers, such as PODXL, WT1 (glomerular podocyte marker), LTL (proximal tubule marker), E-cadherin, and AQP2 (collecting duct marker), which validate their structural and functional properties.

In terms of functional assessment, researchers typically use methods such as albumin filtration, glucose reabsorption, cation transport, and drug toxicity responses to validate organoid performance. For example, the classic cisplatin (cisplatin)-induced nephrotoxicity assay has been used to assess the drug response capacity of kidney organoids (28). However, current kidney organoids still face limitations, including insufficient maturity, poor long-term culture stability, and the absence of a complete glomerular filtration membrane and vascular system. These challenges represent the core areas of focus for optimization and breakthroughs in the field.

### 2.3 Applications of organoids in renal disease pathogenesis research

Kidney organoids offer a powerful tool for investigating the pathogenesis of renal diseases. In the study of genetic kidney disorders, organoid models can be constructed to simulate diseases by combining gene-editing technologies. For instance, using the CRISPR/Cas9 system to edit genes in human pluripotent stem cells, organoids can be generated to model autosomal dominant polycystic kidney disease (ADPKD), enabling the study of cyst formation and related pathological mechanisms (29). In renal cancer research, kidney organoids retain the heterogeneity of primary tumors and can mimic their spatial organization and function, providing a more reliable model for studying renal cancer pathogenesis and developing anti-cancer therapies (30).

In drug nephrotoxicity studies, kidney organoids can replicate histological characteristics similar to natural kidneys in models of acute renal tubular injury induced by cisplatin. After cisplatin treatment, organoids exhibit cellular death, degeneration, and DNA damage, which can be used to predict drug-induced nephrotoxicity (31). Additionally, by investigating the effects of patient plasma on kidney organoids, models of diseases such as primary focal segmental glomerulosclerosis (pFSGS) can be simulated, offering new approaches for studying disease activity and recurrence risk (32).

Kidney organoids have proven to be powerful tools for recapitulating the pathological features of a wide range of renal diseases, from monogenic disorders to acquired injuries. By integrating patient-derived cells, genome editing, and tailored culture conditions, these models can reproduce disease-specific phenotypes and enable mechanistic investigations, drug testing, and therapeutic evaluation in a human-relevant context. Table 2 compiles representative examples of kidney organoid–based disease models, detailing the modeling strategies, phenotypic features, functional readouts, advantages, and limitations for each condition, thereby illustrating the breadth of applications and the translational potential of this technology in nephrology research.

**Table 2:** Applications of kidney organoids in modeling various renal diseases.

Disease type	Modeling strategy	Organoid features simulated	Functional readouts	Advantages	Limitations	Key references
Adult autosomal dominant polycystic kidney disease	CD24^+^ cell-derived adult kidney organoids; CRISPR/Cas9 PKD1/PKD2 knockout; tolvaptan treatment	Adult tubular epithelium; cyst formation mimicking ADPKD	Cyst size measurement; scRNA-seq; drug response	Adult origin, stable long-term culture; recapitulates ADPKD phenotype; suitable for drug testing	Limited nephron complexity; lacks vasculature and stroma; narrow cellular origin	Xu et al. (84)
Alport syndrome	iPSC-derived kidney organoids expressing type IV collagen α5; patient iPSCs vs. mutation-corrected controls; chemical chaperone (4-PBA) treatment	Glomerular basement membrane composition; differential collagen α5(IV) presence	Immunostaining for collagen α5(IV); comparison of mild vs. severe case organoids; treatment response to 4-PBA	Recapitulates GBM defects; distinguishes mild vs. severe phenotype; allows drug testing in human context	Organoids remain immature; glomeruli lack vasculature; correction works only in vitro and in mild cases	Hirayama et al. (34)
Acute kidney injury	Adult rat kidney stem cell (KS cell)–derived kidney organoids; exposed to Beni-koji CholesteHelp supplement vs. cisplatin	Tubular-like and glomerulus-like structures; epithelial cell integrity	Histology: epithelial thinning; cleaved caspase-3 apoptotic marker levels	Rapid, reproducible nephrotoxicity screening platform; mimics tubular injury	Derived from rat, not human; limited nephron complexity; glomerular functionality not assessed	Nakanoh et al. (85)
Lupus nephritis	DNA-CAAR-T cells (six variants against α-actinin, HS, histone-1, C1q) co-cultured with IFN-α-stimulated anti-dsDNA^+^ B cells in human kidney organoids	Immune-mediated kidney damage: altered morphology, apoptosis, inflammation in organoid	Organoid morphology; apoptotic markers; inflammatory cytokines (IFNγ); B-cell killing assays	Demonstrates antigen-specific elimination of pathogenic B cells, protects organoids from damage	In vitro co-culture only, lacks in vivo validation; some specificity controls not fully addressed	Solé et al. (86)
Drug-induced nephrotoxicity	hPSC-derived multi-segmented kidney organoids with integrated ATP/ADP biosensor; exposure to nephrotoxins	Organized nephron-like structures (proximal tubule, podocyte, loop segments); functional OAT1/3 and OCT2	Real-time ATP/ADP ratio changes via biosensor; histology for tubular injury; transporter inhibition rescue assays	Real-time metabolic readout for live monitoring; enables segment-specific toxicity; drug transporter functionality present	Organoids immature; limited efflux transporter expression in young organoids; lacking vasculature and full filtration	Susa et al. (87)
Nephronophthisis	Patient-derived and gene-edited hiPSCs lacking NPHP1, differentiated into kidney organoids; also overexpression rescue	Primary cilia morphology; cysts in medullary-like tubular structures	Cyst formation frequency in suspension organoids; cilia length/morphology; transcriptome of cilia- and cell cycle genes	Human disease model for NPHP1-associated ciliopathy; recapitulates cystogenesis and ciliary defects; isogenic rescue confirms specificity	Organoids remain immature; no vasculature or filtration; fibrosis induction required for further pathology modeling	Arai et al. (88)
Autosomal recessive polycystic kidney disease (ARPKD)	Patient-derived or gene-edited hPSC/iPSC-derived kidney organoids integrated with organ-on-a-chip platforms	collecting duct/distal nephron cystogenesis, mechanosensitive disease phenotypes	cyst formation under flow, pathway analysis, drug-response testing	captures ARPKD-relevant cystogenesis in a dynamic microenvironment; suitable for mechanistic studies and therapeutic screening	incomplete maturation; still lacks full systemic physiology	Kuraoka et al. (36), advanced science

#### 2.3.1 Construction and study of genetic kidney disease models

Kidney organoids provide a reliable platform for studying monogenic hereditary kidney diseases. Researchers can introduce specific mutations at the iPSC level using gene-editing technologies such as CRISPR/Cas9 or directly utilize patient-derived iPSCs to generate organoids, thereby reproducing disease phenotypes in three-dimensional structures.

Examples include:

- *Polycystic kidney disease (PKD):* By knocking out the PKD1/PKD2 genes, cyst-like structures can be observed in organoids, simulating the pathological process of tubular dilation and serving as a platform for drug screening (33).
- *Alport syndrome*: This model simulates defects in the basement membrane and reproduces podocyte pathology, enabling the screening of small molecules that promote collagen repair (34, 35).
- *Autosomal recessive polycystic kidney disease (ARPKD)*: In addition to ADPKD, ARPKD has also been modeled using kidney organoid-on-a-chip systems, which better reproduce flow-dependent cystic dilation and mechanosensing-related disease phenotypes under dynamic culture conditions, thereby providing a valuable platform for mechanistic studies and therapeutic screening (36).
- Fabry disease (37) and other rare genetic diseases (38) are also progressively modeled and studied in organoid systems, advancing the development of precise diagnostic approaches.

#### 2.3.2 Drug toxicity and injury models for acute kidney injury (AKI)

Kidney organoids are ideal models for evaluating drug nephrotoxicity and toxicity related to environmental exposure. Traditional animal experiments cannot fully capture the human kidney’s response differences, whereas organoid models can replicate multiple pathological features of AKI and facilitate high-throughput screening. Common applications include:

- *Cisplatin nephrotoxicity model*: Inducing oxidative stress and proximal tubule injury in organoids to study antioxidant treatment strategies (28).
- *Vancomycin and statin toxicity*: Assessing drug-induced mitochondrial dysfunction and reabsorption impairment (39).
- *Reperfusion injury and hypoxia models*: Combining hypoxic culture with ROS stimulation to simulate the complex mechanisms of AKI onset in clinical settings (40).

Additionally, the degree of injury and recovery can be dynamically assessed using marker expression (such as KIM-1, NGAL) and functional indicators (glucose uptake, electrolyte transport) (41, 42).

#### 2.3.3 Preliminary exploration of chronic kidney disease and fibrosis models

CKD is often accompanied by persistent inflammation and renal interstitial fibrosis. Recent studies have begun to utilize kidney organoids to recapitulate the fibrotic progression induced by chronic stimuli. For example, Davis et al. (43) employed single-cell multiomics to reveal that TGF-β1 drives myofibroblast differentiation via chromatin co-localization of SMAD3 and EZH2, and that the EZH2 inhibitor GSK343 can block this process. Similarly, Yang Xiaoping and colleagues demonstrated that in human iPSC-derived kidney organoids, the bile acid receptor agonist INT-767 reverses TGF-β1-induced fibrosis by activating the farnesoid X receptor and inhibiting the p-SMAD3/TAZ signaling pathway (44). These two studies collectively elucidate the epigenetic regulatory mechanisms of TGF-β signaling in renal fibrosis and identify potential therapeutic targets. Such findings deepen our understanding of TGF-β-mediated fibrogenesis and highlight the application potential of organoids in precision medicine and drug development, offering novel strategies for CKD treatment.

Additionally, co-culture models of organoids with immune cells—such as macrophages and T cells—are under investigation, aiming to simulate the inflammatory microenvironment and its impact on CKD progression.

#### 2.3.4 Personalized disease modeling and precision therapy exploration

Patient-derived iPSC-based “personalized kidney organoids” enable the *in vitro* recapitulation of individual-specific pathological processes and serve several purposes:

- *Predicting patient-specific drug responses*: For instance, Ma et al. (45) developed a proximal tubule chip based on hiPSC-derived kidney organoids that demonstrates excellent capability in modeling physiological drug transport and nephrotoxicity. This system effectively evaluated compounds including adefovir, rosuvastatin, metformin, AA1, and cisplatin, underscoring its potential in drug screening and toxicity assessment and paving the way for personalized models in renal transport and disease modeling.
- *Guiding pathogenicity assessment of rare mutations*: Genetic kidney diseases constitute a major cause of CKD. Although DNA diagnostic techniques have improved detection rates, research into disease mechanisms and drug development remains constrained by the limitations of animal models and scarcity of clinical samples. hiPSC-derived kidney organoids provide a novel platform for genetic disease research, enabling efficient disease phenotype modeling and mechanistic exploration when combined with CRISPR/Cas9 genome editing (46).
- *Customizing small molecule drugs, gene therapy, or mRNA delivery strategies*: Gupta et al. (47) utilized human stem cell-derived kidney organoids to evaluate the toxicological risks of adeno-associated virus (AAV)-mediated CRISPR genome editing. Their results showed that high-titer AAV exposure induces genotoxicity, immunogenicity, and senescence phenotypes in renal tubular cells, activating the classical NF-κB signaling pathway (IL-1β/p21-dependent), which leads to fibrotic damage. Inhibition of NF-κB markedly reduced tubular senescence and fibrosis, suggesting a potential strategy to mitigate clinical risks associated with genome editing therapies. This study validates kidney organoids as efficient preclinical models for assessing AAV-related toxicity and provides a basis for optimizing gene therapy safety.

By integrating personalized organoid models with genomic and transcriptomic data, a “disease-model-treatment” closed-loop system can be established within the framework of precision medicine, propelling renal disease research into a new era of systems integration.

## 3 Applications of organoids in clinical practice of kidney diseases

Kidney organoids have emerged as versatile platforms enabling a broad range of biomedical applications. As illustrated, they are employed in developmental biology to study nephrogenesis (48), disease modeling of congenital and acquired renal disorders (49), and drug screening to evaluate nephrotoxicity and therapeutic efficacy (50). Organoids also contribute to immune response studies, especially for investigating immune–kidney interactions (51), and are promising tools for bioartificial kidney systems via integration with microfluidic platforms (52). Furthermore, kidney organoids support precision medicine by recapitulating patient-specific phenotypes for personalized therapeutic strategies (53).

**Figure 2:**
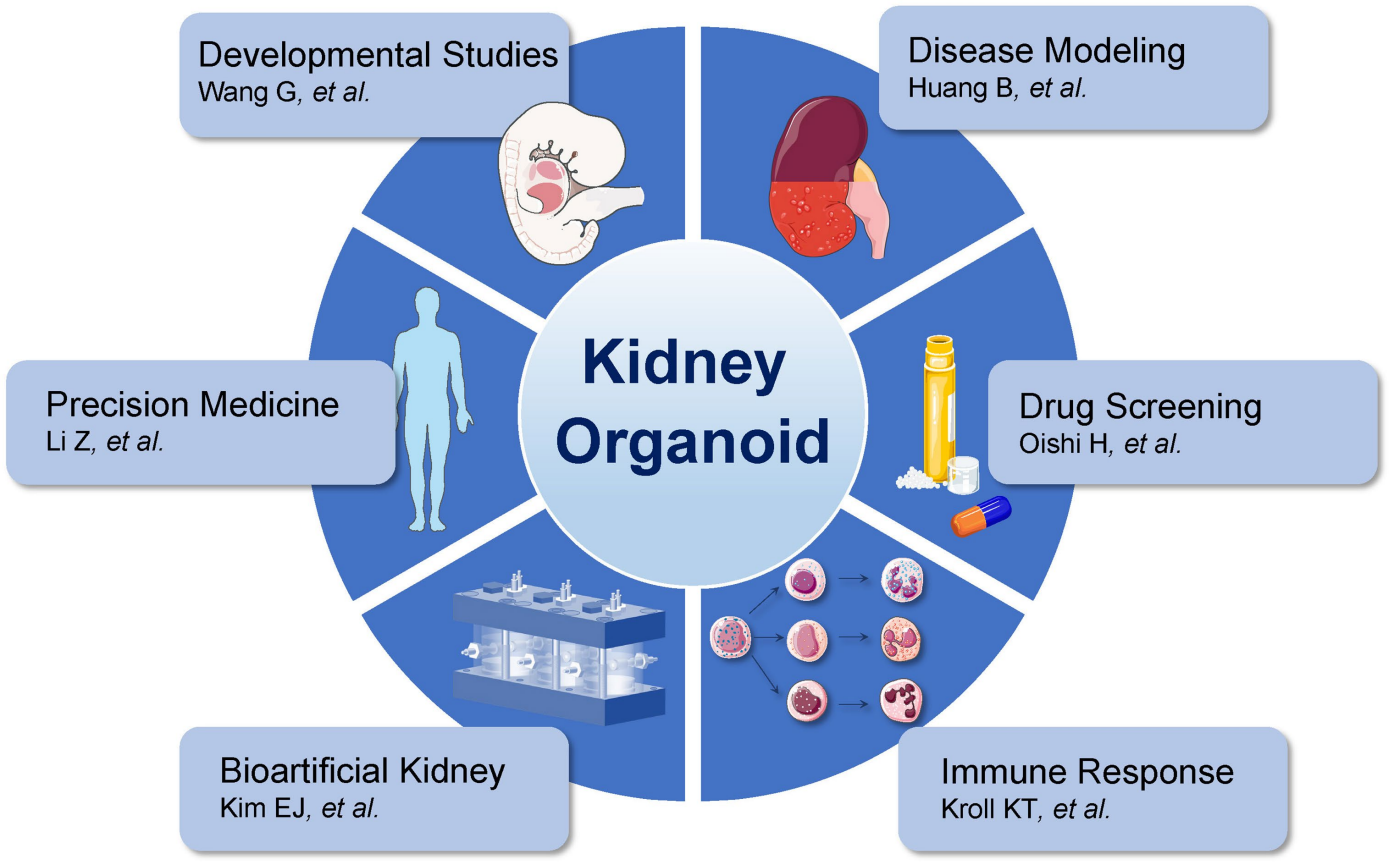
Multifaceted applications of kidney organoids in biomedical research.

Kidney organoids have evolved from experimental models of nephrogenesis into versatile platforms with broad biomedical utility. Beyond recapitulating developmental processes, they enable disease modeling for both congenital and acquired renal disorders, serve as testbeds for nephrotoxicity assessment, and provide innovative systems for studying immune–kidney interactions. Integration with bioengineering technologies further expands their potential in regenerative medicine and precision therapy. To illustrate this spectrum of applications, Figure 2 highlights representative research directions—from developmental biology and disease modeling to drug screening, immune response studies, bioartificial kidney systems, and patient-specific therapeutic strategies—underscoring the multifaceted role of kidney organoids in advancing kidney disease research and clinical translation.

### 3.1 Application of organoids in diagnostic technologies for kidney diseases

Kidney organoids are most relevant to diagnosis when they reproduce patient-specific disease phenotypes or support functional drug-response testing, particularly in inherited kidney disorders and renal cancer. For genetic kidney disorders, patient-derived iPSCs can be used to generate kidney organoids that recapitulate disease phenotypes, thereby assisting diagnosis. For example, in certain hereditary kidney diseases, gene editing of iPSCs to introduce pathogenic mutations enables the construction of kidney organoids exhibiting abnormal development and function, facilitating the clarification of genetic mechanisms and improving diagnostic accuracy (54).

In contrast to hPSC/iPSC-derived kidney organoids used to model developmental and non-neoplastic renal disorders, renal tumor organoids are tissue-derived platforms established directly from patient tumor samples. In renal cancer diagnosis, tumor-derived kidney cancer organoids preserve tumor heterogeneity and microenvironmental features, allowing drug sensitivity testing to guide precision clinical therapy. Studies demonstrate that drug screening on renal cancer organoids can predict patient responses to various anticancer agents, enhancing treatment outcomes (30). Moreover, kidney organoids can be utilized to assess drug-induced nephrotoxicity. In models of cisplatin-induced acute kidney injury, organoids reveal histological and molecular changes indicative of nephrotoxic effects, providing valuable information for clinical medication safety evaluation (55).

### 3.2 Innovations in therapeutic strategies for kidney diseases using organoids

In therapeutic research, kidney organoids are currently most useful for preclinical drug screening, target validation, and evaluation of regenerative strategies. In drug development, kidney organoids can mimic the human renal microenvironment for drug screening and efficacy evaluation. Testing a large library of compounds on kidney organoids enables rapid identification of candidates with potential therapeutic benefits, thus improving the efficiency of drug discovery. For example, kidney organoids have been employed to study the regulatory effects of novel small molecules on kidney disease–related signaling pathways, offering a foundation for targeted therapy development (56).

In regenerative medicine, kidney organoids hold promise as a new avenue for renal replacement therapies. Although clinical application remains a future goal, optimizing organoid construction and culture methods to enhance renal functionality may enable their use in kidney tissue repair and regeneration. Integration with biofabrication technologies, such as 3D bioprinting, facilitates the generation of vascularized kidney tissues, improving organoid viability and function (57). Concurrently, gene editing technologies can be applied to modify organoids to reduce immune rejection, providing improved donor sources for kidney transplantation.

### 3.3 Potential of organoids in personalized medicine for kidney diseases

Patient-derived kidney organoids provide a platform for individualized disease modeling and ex vivo therapeutic testing. By obtaining somatic cells from patients (e.g., skin fibroblasts), reprogramming them into iPSCs, and subsequently inducing kidney organoid formation, patient-specific renal disease features can be modeled *in vitro* (58). These organoids enable personalized drug screening, allowing selection of the most suitable therapeutic agents based on individual organoid responses, thereby enhancing treatment precision and efficacy.

In oncology, however, it is important to distinguish renal tumor organoids from iPSC-derived kidney organoids. Unlike iPSC-derived models generated through directed differentiation, renal tumor organoids are established directly from patients’ tumors and are primarily used to preserve tumor-specific biological characteristics and predict sensitivity or resistance to anticancer drugs, thereby supporting personalized treatment selection (53). Additionally, for certain genetic kidney diseases, patient-specific kidney organoids facilitate in-depth mechanistic studies and provide a basis for developing individualized gene therapy strategies. For instance, gene editing-based correction of pathogenic mutations in kidney organoids derived from affected individuals can be explored as novel therapeutic approaches for hereditary kidney disorders.

## 4 Epidemiological research of kidney diseases using organoids

### 4.1 Application of organoids in epidemiological data analysis of kidney diseases

By investigating organoids derived from diverse populations and geographic regions, the roles of genetic and environmental factors in the onset and progression of kidney diseases can be elucidated. For instance, gene expression profiling of kidney organoids from different ethnic groups can reveal race-associated genetic susceptibility differences, providing a scientific basis for tailored prevention and treatment strategies (13).

In CKD epidemiology, kidney organoids can be used to model disease progression *in vitro*. Coupled with large-scale epidemiological datasets, this approach enables deeper analysis of CKD risk factors and mechanistic pathways. Comparative studies between organoids derived from CKD patients and healthy controls can identify key signaling pathways and molecular targets linked to CKD development, guiding novel therapeutic approaches.

### 4.2 Role of organoids in studying characteristics of dialysis patient populations

For dialysis patient cohorts, kidney organoids can be employed to investigate individual variability and population-level features. Studies on organoids derived from dialysis patients facilitate analysis of how genetic background, lifestyle, and other factors influence dialysis efficacy and complication incidence. For example, alterations in the gut microbiome of dialysis patients have been associated with dialysis-related complications. By constructing kidney organoids from dialysis patients and simulating the effects of gut microbiota metabolites, the interplay between the microbiome, kidney disease, and dialysis complications can be explored in depth (59).

Moreover, kidney organoids can assess individual differences in drug metabolism and responses among dialysis patients. Patients often exhibit variable reactions to medications administered during dialysis; drug sensitivity testing on patient-derived organoids helps elucidate personalized pharmacokinetics and dynamics, providing a basis for individualized drug regimens.

### 4.3 Value of organoids in identifying risk factors for kidney diseases

Kidney organoids assist in identifying risk factors associated with kidney diseases. In studies of pFSGS, exposure of kidney organoids to patient plasma induced pathological changes such as podocytopathy, extracellular matrix protein deposition, fibrosis, and apoptosis, suggesting the presence of circulating risk factors in patient plasma that contribute to pFSGS pathogenesis (32).

Regarding CKD risk factor identification, organoid-based research has shown that inflammation, oxidative stress, and other factors can impair organoid structure and function, closely correlating with CKD onset and progression. Furthermore, metabolomic analyses of kidney organoids have revealed metabolite alterations associated with CKD risk, offering potential biomarkers for early CKD risk detection.

## 5 Challenges in kidney disease research and application using organoids

This table compares three representative culture systems for kidney organoids. *Hydrogel-based static culture* supports 3D organization via extracellular matrix mimicry but suffers from limited nutrient diffusion and batch variability (60). *Microfluidic-based dynamic culture* enables precise spatial confinement and exposure to shear stress, promoting functional maturation and compatibility with metabolic sensing—ideal for disease modeling and drug screening (61). *Bioreactor-based dynamic culture* enhances gas/nutrient exchange and supports long-term, large-scale culture with improved size uniformity, though mechanical stress in low-shear zones remains a challenge (62).

Optimizing the culture environment is pivotal for improving the structural fidelity, functional maturity, and scalability of kidney organoids. Various culture systems—ranging from static hydrogel embedding to dynamic microfluidic and bioreactor-based platforms—have been developed to address limitations such as nutrient diffusion constraints, size heterogeneity, and long-term viability. Each system offers distinct advantages and trade-offs in terms of microenvironmental control, maturation potential, and compatibility with downstream analyses. Table 3 compares these representative culture approaches, outlining their core principles, benefits, and limitations, thereby providing a framework for selecting and refining culture strategies in kidney organoid research.

**Table 3:** Kidney organoid culture systems.

Culture system	Organoid status	Features
Hydrogel-based static culture	Spherical structures of varying sizes embedded or adhered within the hydrogel matrix.	Provides 3D spatial support for self-organization. Mimics cell–ECM interactions. Limited nutrient and oxygen diffusion; central necrosis may occur. Easy to operate but subject to batch variability.
Microfluidic-based dynamic culture	Aligned or confined within microchannels or chambers; exposed to shear stress from fluid flow; enhanced surface polarization.	Simulates vascular or urinary flow, promoting functional maturation. Allows integration with real-time metabolic sensors. Highly controllable environment, ideal for disease modeling and drug screening. More complex to fabricate but enables high-throughput and precise regulation.
Bioreactor-based dynamic culture	Freely suspended in rotating or perfused medium; generally uniform in size and morphology.	Excellent gas and nutrient exchange. Promotes homogeneity and long-term maintenance. Suitable for large-scale and extended culture. Low-shear zones may be hard to control; risk of mechanical stress.

### 5.1 Ethical issues in kidney organoid research

Research involving organoids raises a series of ethical concerns. Regarding cell sources, the use of ESCs sparks controversy over the moral status of embryos. Although iPSCs circumvent the ethical issues associated with ESCs, the reprogramming process of iPSCs may introduce genetic mutations and other potential risks that could affect the safety and reliability of organoids (63).

Furthermore, ethical considerations extend to the application of organoid research outcomes. When organoid technology is used for therapeutic purposes, ensuring treatment safety and efficacy, as well as balancing therapeutic benefits against potential risks, remain critical ethical challenges. Additionally, the collection and use of patient-derived samples in organoid research must comply with stringent ethical guidelines to protect patients’ informed consent rights and privacy.

Since organoids are primarily established from patient tissues or stem cells, issues related to patient privacy protection, informed consent, and ethical review impose significant regulatory constraints. The difficulty of large-scale cell sourcing further complicates clinical translation. Moreover, the presence of animal-derived components in culture systems and associated biosafety concerns present additional barriers to clinical application. Establishing comprehensive ethical and legal frameworks, developing animal-free culture media, and exploring alternative cell sources are essential prerequisites to advancing the clinical translation of kidney organoid technologies.

### 5.2 Technical bottlenecks in therapeutic applications of organoids

The main technical barriers to therapeutic translation of kidney organoids include insufficient maturation, limited vascularization, poor scalability, and substantial batch-to-batch variability. The maturity and functional completeness of kidney organoids require significant improvement. Although current kidney organoids can replicate certain renal functions, they still lag behind native kidneys in terms of cell type diversity, tissue architecture, and functional complexity, which limits their therapeutic efficacy in renal diseases (64).

Vascularization remains a major obstacle. The lack of effective vascular networks restricts nutrient delivery and metabolic waste removal within organoids, impairing their ability to maintain normal functions over extended periods. Additionally, large-scale production and quality control of organoids pose substantial technical difficulties. Developing standardized culture and differentiation protocols to ensure organoid consistency and stability is critical for their broad therapeutic application in kidney disease.

The issues of scalability and standardization are particularly pressing. Currently, kidney organoids are predominantly generated via labor-intensive manual methods with low throughput, hindering scalable manufacturing. The absence of unified preparation standards and quality control systems results in significant variability both across different laboratories and between batches within the same laboratory. To facilitate clinical translation, automated and high-throughput production platforms must be developed, alongside rigorous quality assessment criteria to guarantee batch-to-batch consistency and reproducibility.

### 5.3 Regulatory challenges in kidney disease and treatment using organoids

Regulatory translation of kidney organoids is constrained by the absence of standardized quality criteria, safety assessment frameworks, and clearly defined approval pathways for organoid-based products. At present, quality standards and evaluation systems for organoid-based products remain underdeveloped, complicating assurance of their safety and efficacy. For example, establishing appropriate clinical trial protocols and approval pathways for organoid-based drug development and therapies represents a significant challenge for regulatory agencies (65).

Moreover, the rapid advancement of organoid technologies has outpaced regulatory policy updates. Regulatory bodies must promptly revise and enhance relevant regulations to accommodate the evolving technical landscape. International regulatory harmonization is also crucial, as disparities in standards among countries and regions could impede cross-border collaboration and broader dissemination of organoid technologies.

## 6 Engineering improvements of kidney organoids

Engineering strategies are increasingly being applied to address major limitations of kidney organoids, including insufficient vascularization, incomplete maturation, limited immune complexity, and poor long-term stability. Current advances in this area mainly involve endothelial integration, bioprinting and organ-on-chip platforms, immune-cell incorporation, and optimized long-term culture systems.

### 6.1 Vascularization strategies

The structure and function of kidney organoids heavily depend on the establishment of a functional vascular system. Incorporating microvascular networks can improve oxygen and nutrient delivery, enhance metabolic waste removal, and promote nephron maturation, thereby increasing the physiological relevance of organoids for disease modeling and translational studies. The prevailing strategies for kidney organoid vascularization include endothelial co-culture, flow-induced activation of endogenous endothelial progenitors, transplantation-assisted vasculogenesis, and genetically engineered endothelial niche induction. These approaches are not only important for improving organoid viability, but also for enabling glomerular capillary formation, endothelial specialization, and more accurate modeling of filtration-associated functions.

Notably, Maggiore et al. (66) developed a gene-inducible endothelial microenvironment that successfully achieved vascularization within human kidney organoids *in vitro* and significantly promoted multilineage maturation together with the emergence of renin-expressing cells. They used inducible ETV2-expressing human iPSCs co-cultured with conventional hiPSC lines to generate kidney organoids with extensive endothelial networks, fenestrated glomerular and venous endothelial subtypes, and improved podocyte maturation. This study is particularly important because it moved vascularization beyond a purely supportive role and showed that endothelial niche engineering can directly reshape lineage maturation and induce functionally relevant renal cell populations.

In addition to genetic endothelial induction, transplantation-based vascularization has provided key mechanistic insight into kidney organoid maturation. Koning et al. (67) showed that after transplantation, human organoid-derived endothelial cells can expand, reorganize into perfused capillaries, and form a chimeric vascular network with host-derived vessels. Importantly, perfused glomeruli progressed toward capillary loop-stage maturation, indicating that vascularization is tightly linked to both epithelial and stromal maturation rather than merely improving nutrient supply. These findings suggest that hemodynamic exposure and endothelial–perivascular interactions are central determinants of kidney organoid functional development.

Recent transcriptomic work has further refined this concept. In a 2025 study, Koning et al. (68) used single-cell transcriptomics to show that transplantation not only preserves organoid endothelial cells, but also promotes vessel growth programs and arterial maturation. Together with the *in vitro* ETV2-induction strategy reported by Maggiore et al. (66), these data indicate that future vascularization efforts should aim not simply to increase vessel number, but to guide endothelial subtype specification, mural cell recruitment, and flow-responsive maturation.

Despite these advances, vascularization of kidney organoids remains incomplete. *In vitro* systems still struggle to generate stable, fully perfusable glomerular capillary beds and a mature arteriolar–capillary–venous hierarchy, whereas transplantation models often rely on host-derived circulation and therefore reduce experimental controllability (69–71). Accordingly, the next phase of the field will likely require integration of endothelial engineering, perfusion-based culture, and biomaterial-guided spatial organization to achieve more physiologically faithful vascular maturation in a fully humanized setting.

### 6.2 Integration of bioprinting and organ-on-chip technologies

#### 6.2.1 Bioprinting-assisted kidney organoid fabrication

Bioprinting technology enables high-precision deposition of cells and biomaterials to fabricate kidney organoids with defined three-dimensional architectures and improved reproducibility. Compared with conventional manual aggregation methods, bioprinting offers stronger control over organoid size, cell number, initial geometry, and spatial arrangement, which is particularly important for kidney organoids because nephron patterning, tissue conformation, and batch-to-batch consistency strongly influence downstream maturation and functional readouts.

Lawlor et al. (23) demonstrated that cellular extrusion bioprinting can automate the fabrication of self-organizing kidney organoids while maintaining morphology, component cell types, and transcriptional identity comparable to manually generated organoids. Importantly, this approach markedly improved reproducibility and enabled rapid generation of organoids in both 6-well and 96-well formats, thereby substantially enhancing throughput and quality control. The authors also showed that the 96-well format was compatible with compound dosing, highlighting the translational value of bioprinting for nephrotoxicity testing and screening applications.

More recently, Shin et al. (72) developed a low-cost, high-throughput customizable 3D bioprinting system for generating human iPSC-derived kidney organoids. Their platform printed nephron progenitor cells to form organoids expressing markers of podocytes, proximal tubules, distal tubules, and endothelial cells, supporting the idea that accessible printing systems may broaden adoption of standardized kidney organoid production. Taken together, these studies indicate that bioprinting is not merely a manufacturing tool; rather, it is becoming a means to improve structural fidelity, scalability, and experimental standardization across kidney organoid workflows (23).

Nevertheless, bioprinting alone does not fully resolve the central limitations of kidney organoids. Although it improves reproducibility and spatial control, printed organoids may still remain functionally immature if they are maintained under static conditions without physiologically relevant perfusion, endothelial remodeling, or biomechanical stimulation. Therefore, the major value of bioprinting in the kidney organoid field lies in providing a structurally standardized starting platform that can subsequently be coupled with dynamic culture systems, vascularization strategies, and biosensing technologies (73, 74).

#### 6.2.2 Organ-on-chip-enabled dynamic conditioning and vascular interaction

When combined with microfluidic organ-on-chip platforms, kidney organoids can be cultured under dynamic perfusion conditions that better mimic renal blood flow, solute exchange, and fluid shear stress. This is particularly important because the kidney is a flow-sensitive organ in which endothelial maturation, tubular polarity, and transport-associated functions are tightly influenced by biomechanical cues. Organ-on-chip systems therefore add a functional layer that static culture and printing alone cannot provide.

For instance, Homan et al. (70) developed a millifluidic culture platform in which fluid flow greatly expanded the endogenous endothelial progenitor cell pool within kidney organoids and generated vascular networks with perfusable lumens surrounded by mural cells. Vascularized organoids cultured under flow displayed more mature podocyte and tubular compartments, enhanced polarity, and more adult-like gene expression compared with static controls. The study further showed that disruption of endogenous VEGF gradients reduced vessel association with nephron compartments, indicating that flow and vascular signaling act together to promote maturation.

Similarly, Bas-Cristóbal Menéndez et al. (69) established a humanized kidney organoid–vasculature interaction model using a microfluidic organ-on-chip system. By co-culturing iPSC-derived kidney organoids with HUVECs, they observed endothelial migration into organoid tissue, interconnection with endogenous endothelial cells, and formation of open-lumen vessel-like structures. This work is significant because it demonstrates that chip-based compartmentalization can be used not only to perfuse organoids, but also to reconstruct endothelial ingrowth and cross-talk in a controllable *in vitro* setting.

Beyond these foundational studies, recent engineering efforts have further emphasized the importance of scalable and maturation-supportive culture environments. Kim et al. (74) developed the UniMat platform, a 3D geometrically engineered permeable membrane system that improves both organoid uniformity and maturity by constraining growth geometry while allowing unrestricted access to soluble factors. Although UniMat is not a classic microfluidic chip, it reinforces the same engineering principle: structural design of the culture environment can profoundly influence organoid consistency, nephron development, vascularization, and long-term stability. This is highly relevant to kidney organoid translation, because dynamic conditioning must ultimately be paired with scalable manufacturing platforms.

Taken together, organ-on-chip systems should not be viewed simply as add-on culture devices. Rather, they provide a mechanistically important platform for introducing flow-dependent maturation, endothelial recruitment, immune cell circulation, and real-time functional interrogation (70). In future applications, the most powerful strategy will likely be the integration of bioprinting-based spatial control with chip-enabled dynamic conditioning, thereby combining reproducible construction with physiologically relevant function.

### 6.3 Immune microenvironment simulation

Kidney diseases often involve immune-mediated inflammatory injury, yet conventional kidney organoid models mainly recapitulate epithelial and stromal compartments while lacking immune cell populations and immune–tissue interactions. This limitation is particularly important for studying autoimmune nephritis, transplant rejection, inflammatory fibrosis, and immune-related nephrotoxicity. Therefore, reconstruction of the immune microenvironment is becoming an increasingly important engineering direction for improving the biological relevance of kidney organoids.

Early work in this area established that immune cells can infiltrate kidney organoids *in vitro*. Shankar et al. (75) described a kidney organoid–PBMC co-culture system in which immune cells infiltrated organoid tissue, demonstrating the feasibility of modeling direct immune–renal interactions. However, the authors also highlighted key limitations of static co-culture, including poor long-term PBMC survival and restricted oxygen supply that contributes to central necrosis in organoids. These observations are important because they show that adding immune cells alone is insufficient; the immune component must be supported by an appropriate tissue-engineering environment.

A more advanced solution was reported by Kroll et al. (51), who developed an immune-infiltrated, vascularized kidney organoid-on-chip model in which peripheral blood mononuclear cells and T cell bispecific compounds were co-circulated under flow. This study demonstrated that dynamic perfusion can support immune cell recruitment and cytotoxic interactions in a far more physiologically relevant context than static culture. Importantly, the model provided a platform for assessing “on-target, off-tumor” toxicities of immunotherapeutics, thereby extending kidney organoids from developmental and disease models into immune safety testing systems.

These studies collectively suggest that the immune microenvironment of kidney organoids should be understood as a multi-parameter system involving immune cell composition, cytokine gradients, endothelial interfaces, and fluid dynamics. Static immune co-culture may be suitable for short-term proof-of-concept studies, but modeling chronic inflammation, immune surveillance, or therapeutic immune injury will likely require vascularized and perfused microphysiological systems. At the same time, major challenges remain, including limited diversity and persistence of immune cell populations, difficulty maintaining immune–epithelial homeostasis, and lack of standardized readouts for immune-mediated tissue injury. Accordingly, future progress in this field will depend on integrating organoid engineering with immunology, vascular biology, and high-content analytical platforms (75, 76).

### 6.4 Long-term maintenance and maturation issues

Long-term culture and functional maturation of kidney organoids remain significant technical challenges. Most organoids can only be maintained for a limited time *in vitro*, with incomplete functional performance, making it difficult to sustain the complex metabolic and endocrine functions of the kidney. Optimizing culture media formulations by adding specific growth factors and extracellular matrix components can promote cell differentiation and tissue remodeling. Mechanical stimuli such as shear stress and cyclic stretching help mimic the physiological kidney environment and promote functional maturation. Combining gene editing and cell engineering technologies also offers new possibilities to enhance organoid stability and functionality. Achieving long-term stable culture of organoids will greatly enhance their experimental and clinical utility, especially in modeling chronic kidney diseases and studying chronic drug toxicity.

For instance, Kim and colleagues proposed an innovative platform called UniMat for large-scale production of uniform and mature kidney organoids, addressing issues of morphological inconsistency and developmental immaturity in traditional methods. The UniMat platform utilizes a 3D geometrically designed permeable membrane that not only provides geometric constraints to ensure uniform organoid growth but also allows free supply of soluble factors to promote maturation. Using UniMat, they successfully generated more consistent kidney organoids in 3D culture, which exhibited higher maturation, including enhanced vascularization, increased nephron-like structures, and improved long-term stability. Furthermore, UniMat was shown to effectively model diseases such as polycystic kidney disease and acute kidney injury, demonstrating its potential for disease modeling and drug screening applications (74).

## 7 Future perspectives of organoids in kidney disease research and therapy

### 7.1 Future development directions of organoid technology in kidney disease research

In the future, organoid technology in kidney disease research will advance toward greater precision and higher efficiency. On one hand, by optimizing organoid construction techniques and integrating cutting-edge technologies such as single-cell sequencing and gene editing, the developmental accuracy and functional integrity of organoids can be further improved. For example, single-cell sequencing can provide in-depth insights into the gene expression profiles of different cell types within organoids, offering a basis for precise regulation of organoid differentiation and development (72).

On the other hand, the combination of organoid technology with organ-on-chip platforms will become an important developmental direction. Constructing organoid models on chips can simulate the complex physiological microenvironment *in vivo*, enabling dynamic monitoring of kidney diseases and high-throughput drug screening. Furthermore, the integration of organoid technology with artificial intelligence is also highly anticipated. Leveraging AI algorithms to analyze large datasets generated by organoids can accelerate drug development, enhance diagnostic accuracy, and improve treatment precision.

### 7.2 Potential breakthroughs of organoids in dialysis treatment innovation

In the field of dialysis treatment innovation, organoid technology is expected to achieve significant breakthroughs. With continuous advancements in organoid construction and culture techniques, it may become possible to develop organoid-based dialysis devices with more complete functions. For example, constructing vascularized kidney organoids with high metabolic activity could enhance the removal of toxins and regulation of electrolyte balance during dialysis, thereby improving treatment efficacy (73).

Moreover, organoid technology can be utilized to develop personalized dialysis treatment plans. By comprehensively analyzing patient-derived kidney organoids, the specific renal function characteristics and disease features of patients can be understood, enabling customization of dialysis parameters and pharmacological regimens tailored to individual patients. This approach aims to improve treatment precision and patient quality of life. Additionally, the integration of organoid technology with advanced materials science may lead to the development of dialysis membranes and devices with better biocompatibility and superior performance.

### 7.3 Prospects for interdisciplinary collaboration in kidney disease research using organoids

The development of organoid technology in kidney disease and dialysis treatment relies heavily on interdisciplinary collaboration. The integration of biology, medicine, engineering, and materials science will drive innovative advances in organoid technology. For instance, research in biology and medicine provides the theoretical foundation for organoid construction and disease modeling, while engineering and materials science contribute technological support for organoid culture, vascularization, and functional optimization (64).

In clinical applications, interdisciplinary collaboration facilitates the translation of organoid technology into practical therapeutic methods. Multidisciplinary teams including nephrologists, surgeons, and bioengineers can jointly develop application strategies for organoids in kidney disease treatment and dialysis, enhancing therapeutic outcomes. Furthermore, such collaboration promotes the application of organoid technology in drug development, medical device innovation, and other related fields, advancing the entire landscape of kidney disease management.

Kidney organoids have become an important platform for studying renal development, modeling human kidney diseases, evaluating nephrotoxicity, and exploring patient-specific therapeutic responses. Compared with conventional two-dimensional culture systems and many animal models, they provide a more human-relevant and structurally complex framework for investigating disease mechanisms and preclinical drug effects.

Despite these advances, major barriers remain before kidney organoids can support broader translational or clinical applications. In particular, incomplete maturation, limited vascularization, restricted long-term stability, and insufficient standardization continue to constrain reproducibility and functional fidelity. Future progress will depend on integrating bioengineering approaches, including organ-on-chip systems, bioprinting, biosensing, and controlled microenvironment design, with rigorous quality control and clinically relevant validation strategies.

## Glossary

2D: two-dimensional
hiPSC: human induced pluripotent stem cell
3D: three-dimensional
hPSCs: human pluripotent stem cells
AAV: adeno-associated virus
HUVECs: human umbilical vein endothelial cells
ADPKD: autosomal dominant polycystic kidney disease
iETV2-hiPSCs: inducible ETV2-expressing human induced pluripotent stem cells
AI: artificial intelligence
iPSC: induced pluripotent stem cell
AKI: acute kidney injury
NF-κB: nuclear factor kappa-light-chain-enhancer of activated B cells
ALI: air-liquid interface
NGAL: neutrophil gelatinase-associated lipocalin
cAMP: cyclic adenosine monophosphate
PBMCs: peripheral blood mononuclear cells
CKD: chronic kidney disease
pFSGS: primary focal segmental glomerulosclerosis
CRISPR/Cas9: clustered regularly interspaced short palindromic repeats/CRISPR-associated protein 9
ROS: reactive oxygen species
ECM: extracellular matrix
scRNA-seq: single-cell RNA sequencing
ESC: embryonic stem cell
TCBs: T cell bispecific antibodies
ESRD: end-stage renal disease
TGF-β1: transforming growth factor beta 1
EZH2: enhancer of zeste homolog 2
VEGF: vascular endothelial growth factor
